# The Association of Flow-Mediated Dilatation and Blood Parameters in Primary Raynaud's Phenomenon

**DOI:** 10.1155/2022/9347946

**Published:** 2022-01-31

**Authors:** Süheyla Uzun, İlker Kaya

**Affiliations:** ^1^Department of Internal Medicine, Gaziosmanpasa University, Tokat, Turkey; ^2^Department of Cardiovascular Surgery, Tokat State Hospital, Tokat, Turkey

## Abstract

**Introduction:**

Raynaud's phenomenon (RP) is a multifactorial disorder. If any underlying disease cannot be determined to be responsible for RP, then it is considered to be the primary RP (pRP). We aimed to investigate the differences between laboratory markers and impaired endothelial function in pRP.

**Materials and Methods:**

Forty-two pRP patients and 30 healthy individuals were included as the study and control groups, respectively. The endothelial function was evaluated with flow-mediated dilatation (FMD) of the brachial artery. The blood samples were obtained from both groups, and white blood cell (WBC), hemoglobin, platelets, mean platelet volume (MPV), creatinine, alanine aminotransferase (ALT), aspartate aminotransferase (AST), D-dimer, fibrinogen, albumin, fibrinogen-to-albumin ratio (FAR), neutrophil-to-lymphocyte ratio (NLR), D-dimer-to-albumin ratio (DDAR), and monocyte chemoattractant protein-1 (MCP-1) parameters were studied. The blood parameters and FMD values obtained were compared between groups.

**Results:**

The groups were similar in regard to age, gender, and smoking history (*p* < 0.05). There was no difference between the two groups in regard to hemoglobin, platelet, MPV, creatinine, ALT, D-dimer, albumin, FAR, NLR, and DDAR levels (*p* < 0.05). AST levels were slightly higher in the pRP group (*p*=0.027). Markedly increased WBC, fibrinogen, MPV, and MCP-1 values were detected in the pRP group (*p*=0.001), as well as higher abnormal FMD responses (*p*=0.001). There was a direct correlation between abnormal FMD response and serum MCP-1 values in patients with pRP (*R*: 0.308, *R*^2^: 0.095, *p*: 0.044).

**Conclusion:**

It seems to be that MCP-1 levels are higher in patients with pRP, and increased values of MCP-1 levels seem to be related to impaired endothelial functions.

## 1. Introduction

Raynaud's phenomenon (RP) is a reversible vasospastic disorder that may be provoked by exposure to cold or by emotional stress. It involves extremity arteries and presents with ischemic symptoms in the extremities [1]. RP can be a preliminary finding of some diseases, in which case it is referred to as secondary RP (sRP). The disorder can be related to traumas or microtraumas resulting from prolonged usage of vibratory tools, exposure to some chemicals, and autoimmune rheumatic or connective tissue diseases, such as systemic sclerosis. If it progresses, it can result in digital ulcers or microamputations [1–3]. However, RP can also occur in the absence of underlying disease or unknown etiology, in which case it is referred to as primary RP (pRP). This sort of pRP is commonly linked to atmospheric conditions, and therefore, the symptoms are dramatically relieved when exposure to the cause is terminated [4, 5]. In the primary type, there is no autoimmunity-associated marker or reaction and therefore, a comprehensive anamnesis should be taken and a physical and laboratory examination should be performed in every case of RP, since after excluding suspected pathologies, the RP cases are often classified as primary type [1, 5].

The essential finding of pRP is aggravation of symptoms through exposure to cold and the detection of color changes in the skin, which turns white, blue, or both. The diagnosis is clarified after exclusion of possible underlying diseases, such as checking nails with capillaroscopy or a laboratory panels such as complete blood counts, inflammatory parameters, metabolic panel, antibodies, and enzymes. Avoiding contact with cold and stress is suggested for these patients, and lifestyle changes such as reducing smoking and caffeine use are important. In addition, some medications can be applied [1–5]. Some patients have borderline symptoms without physical findings, similar to a feeling of cold within the extremities. In this patient group, management is more challenging when no finding apart from anamnesis has been determined and there is an absence of parameters for possible detection [6, 7]. In particular, an abnormal capillaroscopic finding for differentiation of RP is discriminative for secondary RP. Thus, nailfold capillaroscopies are losing their role as potential predictors of pRP.

In recent years, changes in blood parameters have been frequently examined to better define or differentiate borderline or suspected diseases. In particular, inflammatory biomarkers have become quite popular. Investigation of mean platelet volume (MPV) and neutrophil-to-lymphocyte ratio (NLR) levels is a good example of these current studies. NLR has also become an inflammatory predictor that is currently used in clinics. It has been predicted that NLR may be a valuable marker in many systemic inflammatory processes [8]. Moreover, it has been emphasized that these parameters may be predictive in vascular diseases. While MPV values are predicted to have a diagnostic predictive value in patients with thromboangiitis obliterans involving the distal vascular bed, which is a disease very similar to RP, on the other hand, NLR values were also found to be associated with intima-media thickness in patients with Behcet's disease [9, 10]. In addition, monocyte chemoattractant protein-1 (MCP-1) is a serum inflammation parameter that has been investigated in similar conditions and it has been less studied in vascular system diseases compared to other parameters [11].

The aim of this study was to investigate the possible predictors for pRP. In the scope of this objective, flow-mediated dilatation (FMD), routine blood markers, and MCP-1 levels were compared between pRP patients and healthy individuals.

### 1.1. What Is Already Known about This Topic?

Raynaud's phenomenon (RP) is a reversible vasospastic disorder that may be provoked by cold or emotional stress. This phenomenon can occur without underlying disease or unknown etiology, in which case it is referred to as primary RP (pRP). The essential finding of pRP is an aggravation of symptoms by exposure to cold and the detection of skin color changes in the extremities (turns white, blue, or both). The diagnosis is clarified after exclusion of possible underlying diseases by checking nail capillaroscopy and laboratory panels, such as complete blood counts, inflammatory parameters, metabolic panel, antibodies, and enzymes. However, any specific laboratory marker has not been determined for diagnosing pRP. Therefore, researchers have tried to establish an objective scientific laboratory marker that is highly specific to pRP.

### 1.2. What This Study Proposes?

To the best of our knowledge, this is the first study examining the relationship between FMD response (impaired endothelial response) and blood parameters, in patients with pRP. This is also the first report regarding the relationship between impaired endothelial response and MCP-1 levels in pRP. Our results have indicated that patients with pRP have a higher impaired endothelial response to ischemia and the incremental levels of serum WBC, MPV, MCP-1, and fibrinogen values were detected in the pRP group. Moreover, a moderate direct correlation was found between abnormal FMD response and serum MCP-1 values.

## 2. Materials and Methods

The study steps were designed according to the Declaration of Helsinki, and ethical approval was obtained from the Local Ethics Committee of the University. Signed informed consent was obtained from all participants. This study was designed as a prospective, case-control study.

**Figure 1:**
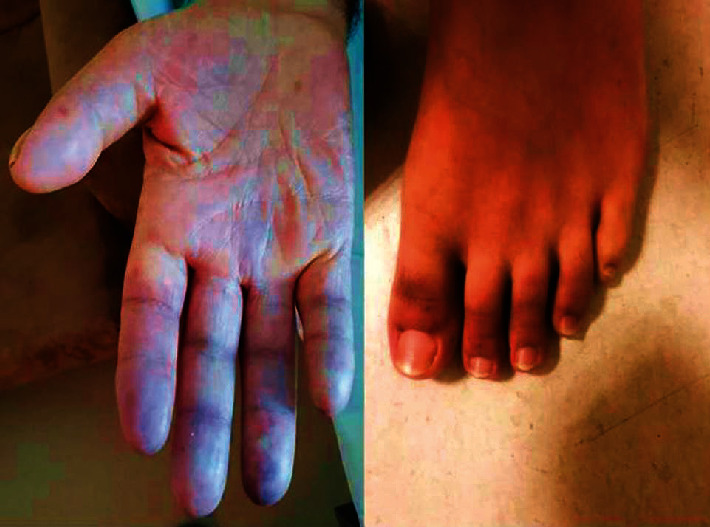
Bruising on the hand and foot in patients with pRP.

Patients over 50 years of age (because of increased risk of atherosclerotic occlusive arterial diseases), patients with a past medical history with trauma, thoracic outlet syndrome, secondary RP (malignity, vasculitis, systemic inflammatory disease, autoimmune diseases, etc.), and anti-inflammatory or corticosteroid usage, and patients with accompanying diabetes, hypertension, and familial hyperlipidemia were all excluded from the study. After exclusion from secondary RP, a total of 43 patients were included, who admitted to the cardiovascular surgery clinic with RP symptoms (bruising and cold hands or feet, provoked by cold) and were diagnosed as primary RP (Figure 1). The control group was formed from healthy individuals who admitted to the hospital for a routine checkup, without cardiovascular symptoms.

### 2.1. Evaluation of Flow-Mediated Dilatation (FMD)

The FMD evaluation was performed on each participant, in the early morning after an overnight fasting period in a standard ultrasound room by an expert sonographer, before starting other steps of the study (blood sampling), the whole as prescribed in the literature [12, 13]. An Android-compatible linear transducer (Philips Lumify L12-4 (12–4 mHz); Philips Ultrasound Inc., Bothell, WA) was used to perform evaluation of vessel diameters. The normal diameter of the brachial artery was measured over approximately 5 cm of the antecubital fossa, and thereupon, the evaluation point was marked. The sphygmomanometer cuff was placed to the proximal site of the evaluation point and inflated to above 50 mmHg of normal blood pressure for five minutes, as prescribed in the literature [12, 13]. After 5 minutes of ischemia period, the cuff was deflated and the ultrasound probe was placed on the marked point, for measurement of postischemic diameter of the brachial artery. The enlargement of diameter equal or above 5% was accepted as a normal response, whereas diameter lower than 5% was accepted as an abnormal vascular response to ischemia, indicating an abnormal endothelial function [12, 13].

### 2.2. Blood Sampling

After evaluation of the FMD response, the routine 10 ml venous blood samples were obtained from each participant. Following centrifugation, routine laboratory parameters (white blood cell (WBC) (10^−3^/uL), hemoglobin (g/dL), platelet (10^−3^/uL), mean platelet volume (MPV) (fL), creatinine (mg/dL), alanine aminotransferase (ALT) (IU/L), aspartate aminotransferase (AST) (IU/L), D-dimer (ng/L), fibrinogen (*μ*g/ml), albumin (g/dL), fibrinogen-to-albumin ratio (FAR) (%), neutrophil-to-lymphocyte ratio (NLR) (%), D-dimer-to-albumin ratio (DDAR) (%), and monocyte chemoattractant protein-1 (MCP-1) (pg/ml) levels were studied from the acquired serum samples. The commercially available human MCP-1 ELISA kit (SUNLONG, Sun Long Biotech Co., LTD) was used for determining MCP-1 levels (SUNLONG) as described in previous reports [11].

### 2.3. Statistical Analysis

In this study, sample collection calculation was made with power analysis by using GPower 3.1.9.4 program to calculate sample size and *α* = 0.05, *β* = 0.10, and effect size = 0.776 were chosen. Since the MCP-1 parameter was thought to be the most decisive data in the study, these values from the reference article were used to calculate the effect size [14]. The nominal data analysis was made using the Yates-corrected chi-square test. A comparison of parametric data between the two groups was made using Student's *t*-test. The Pearson correlation coefficients were used to compare the relation of parameters with FMD. The SPSS 17.0 (SPSS Inc., Chicago, IL, USA) statistical software program was used for all statistical analysis, and a *p* value less than 0.05 was considered significant. By utilizing receiver operating characteristic (ROC) analysis, the area under the curve (AUC) with lower and upper bounds was used to calculate the cutoff value, specificity, and sensitivity. In order to make a Raynaud phenomenon predictive analysis with abnormal FMD, WBC, and MCP-1 values, the cutoff values obtained in the ROC analysis and the variables were categorized and a multiple binary regression analysis was performed. Nagelkerke's *R*^2^ determination coefficient was used to evaluate the predictive ability of the model [15].

## 3. Results

The groups were similar (*p* > 0.05) in regard to demographical variables (age, gender, and smoking history), but markedly higher abnormal FMD response rates were detected in the primary RP patient group (*p*=0.001). The comparison of demographical, clinical (FMD), and laboratory variables between control and pRP patients is demonstrated in Table 1.

Serum hemoglobin, platelet, creatinine, ALT, D-dimer, FAR, NLR, albumin, and DDAR values were found to be similar between the groups (*p* > 0.05). Slightly higher AST values (*p*=0.027) were detected in the pRP group (25.60 ± 9.77 IU/L), when compared with the healthy subjects (21.20 ± 5.14 IU/L). Serum WBC, MPV, fibrinogen, and MCP-1 values were significantly higher in the pRP group (Table 1), when compared with the healthy control group (*p* < 0.05).

**Figure 2:**
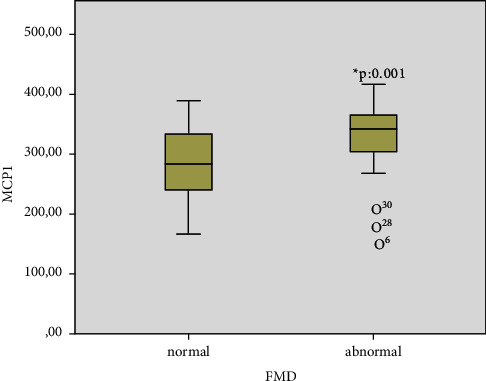
Comparison of monocyte chemoattractant protein-1 (MCP-1) values in normal and abnormal flow-mediated dilatation (FMD) groups.

Higher MCP-1 values (Figure 2) were detected in participants with an abnormal FMD response (*p*=0.001). A significant correlation was detected between abnormal FMD responses and MCP-1 levels (R: 0.308, R^2^: 0.095, *p*: 0.044). In addition, a low-level relationship was found between FMD response and MPV, MCP-1, and fibrinogen values (Table 2).

We studied abnormal FMD, WBC, and MCP parameters by using the binary logistic regression model. Nagelkerke's *R*^2^ determination coefficient was used to evaluate the predictive ability of the model. This model correctly predicted a control value of 83.3% and Raynaud's phenomenon of 86.0% (Table 3). The Nagelkerke *R*^2^ value explains 64.3% of the variance to distinguish whether it is Raynaud's or not.

**Figure 3:**
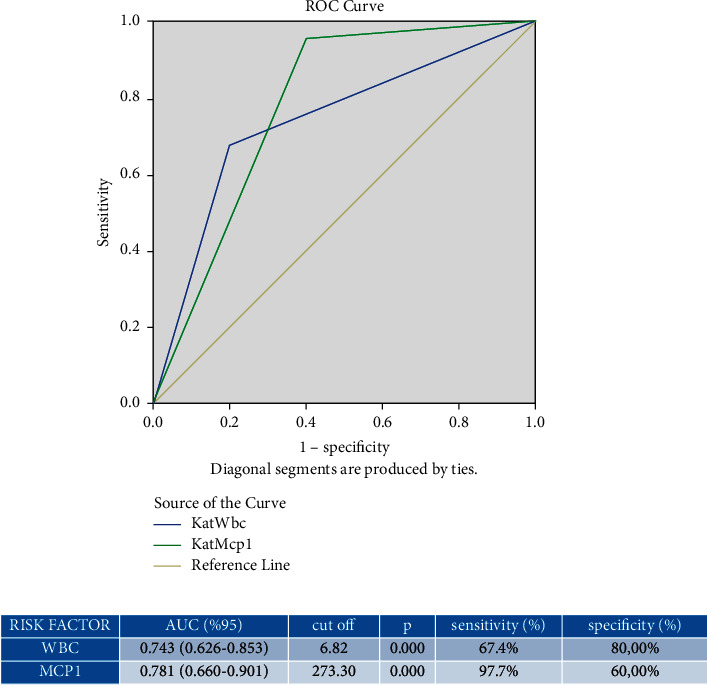
ROC analysis of the serum WBC and MCP-1 values for prediction of primary Raynaud's phenomenon.

When the WBC and MCP-1 values were analyzed using the ROC curve method, the optimum diagnostic cutoff point was found to be 6.82 10^−3^/uL for WBC with a sensitivity of 67.4% and specificity of 80% and the optimum diagnostic cutoff point was found for MCP-1 at 273.30 pg/ml, with a sensitivity of 97.7% and a specificity of 60% (Figure 3).

## 4. Discussion

To the best of our knowledge, this is the first study examining the relationship between FMD response (impaired endothelial response) and blood parameters in patients with pRP. This is also the first report regarding the relationship between impaired endothelial responses and MCP-1 levels in pRP patients. Our results have indicated that patients with pRP have higher impaired endothelial response to ischemia. Incremental levels of serum WBC, MPV, MCP-1, and fibrinogen values were detected in the pRP group. Moreover, a moderate direct correlation was found between abnormal FMD responses and serum MCP-1 values. In multiple binary logistic regression analyses, WBC and MCP-1 were determined to be the most meaningful blood parameters for identifying pRP patients, with acceptable sensitivity and specificity rates.

Some of the predicted parameters in the distinction of pRP and sRP are age and gender. The first symptom attacks usually begin under the age of 30 for pRP and are generally observed more frequently in the female gender. In our study, the mean age of the patient group was found to be 28.33 ± 8.71, which is consistent with the literature, and the female gender was predominant (79%) [16]. The impaired endothelial dysfunction has been demonstrated in both primary and secondary RP in previous studies. It has also been shown that microvascular derangement was associated with endothelial dysfunction in RP patients [17, 18]. The endothelium has an important role in the regulation of vascular response against blood flow-associated shear stress and ischemia-reperfusion events. Insufficient flow-mediated vasorelaxation response is an indicator for increased vascular disease [19]. The dilatation response of the brachial artery after a 4-to-5 minute ischemic period is a reliable, noninvasive assessment method for the evaluation of endothelium functions. The postischemic brachial artery dilatation equal to or greater than 5% of normal diameter is accepted as a normal endothelial function, whereas dilatation with a lower than 5% diameter is accepted as impaired endothelial functions [13, 20].

The FMD response has been studied in patients with RP, albeit on small populations. Flavahan has indicated that an FMD response is normal in pRP and added that cold-induced disruption of arteriovenous connections in RP cases can lead to digital artery spasm [21]. Mavrikakis et al. studied FMD in patients with sRP, and they detected an abnormal FMD response in these patients. In addition, they found that ascorbic acid does not reverse endothelial vasomotor dysfunction in their study [22]. Klein-Weigel et al. investigated the seasonal variations in patients with RP, and they did not show an abnormal FMD response in pRP [23]. Although our results indicated that an abnormal FMD response was detected in 5 (17%) of the 30 healthy individuals, the abnormal FMD was detected in 30 (70%) of the 43 pRP patients.

Routine blood parameters have been investigated for several kinds of vascular diseases [24]. Platelet indices have been investigated in vasospastic disorders detected with a cold stimulation test by Kadan et al. [25]. They found a relationship between the severity of disease and MPV. Shemirani et al. found an independent relationship between pRP and higher serum MPV levels [26]. Another study on higher MPV suggested it was a triggering factor for pRP [27]. In the same study, WBC levels were found to be an insignificant predictor. Lau et al. found increased white blood cell activation in patients with RP [28]. Our own findings supported that MPV and WBC levels are found in patients with pRP. Plasma fibrinogen is another laboratory parameter that was investigated in vasospastic disorders. Splenger et al. concluded that increased plasma fibrinogen levels in patients with RP are related to disrupted distal microcirculation [29]. Similarly, higher fibrinogen levels were found by Żuk et al. to be associated with plasma fibrin clots, displaying impaired lysability and increased endothelial damage in pRP [30]. In our study, higher fibrinogen levels were detected in pRP patients.

MCP-1 is an important potent regulatory chemokine that is responsible for the migration and the infiltration of monocytes. Ischemia, oxidative stress, released cytokines, or growth factors trigger the biological activity of MCP-1 and lead to infiltration of monocytes/macrophages [31]. Rajagopalan et al. investigated MCP-1 levels in sRP and pRP, and they found that MCP-1 levels were higher in sRP, when compared with the primary type. However, they did not compare the MCP-1 levels with healthy controls and did not investigate the relation with endothelial response [31]. MCP-1 was suspected in the pathogenesis of vasospastic outcomes of diseases that presented with sRP [32, 33]. However, the literature includes insufficient reports about the relationship between impaired endothelial function and MCP-1 levels in pRP. Furthermore, another insufficiency exists regarding the differences between pRP and normal populations, in regard to MCP-1 levels. Our results indicate that MCP-1 levels are higher in patients with pRP, and these results reveal a relationship between impaired endothelial functions and serum MCP-1 levels.

In conclusion, our findings demonstrated that MPV, WBC, fibrinogen, and MCP-1 levels were higher in pRP patients, when compared with the healthy control. It appears that increased MCP-1 values were related to abnormal FMD responses in the pRP group. These results might be helpful for the establishment of the pathophysiology of the RP.

### 4.1. Limitations of the Study

The main limitation of the study is concerning the small sample size. Although previous studies have presented findings with smaller patient groups, larger comprehensive cohorts are required to obtain compelling and precise results. The second limitation is concerning the determination of endothelial response with a single method (FMD). The endothelial response should be confirmed with other techniques in order to present clear findings. The third limitation is related to the nature of the disease: if no underlying disease is determined to be responsible for RP, then it is called primary RP. However, there is a multifactorial etiology for RP, and it is therefore possible to omit a proper diagnosis.

## Figures and Tables

**Table 1:** Comparison of demographic and laboratory variables in pRP and healthy control groups.

Variables	Control (*N*: 30)	Raynaud's phenomenon (*N*: 43)	*p* ^#^
Female, *n* (%)	22 (73%)	34 (79%)	0.772
Age (mean ± SD)	27.17 ± 8.01	28.33 ± 8.71	0.565
Smoking, *n* (%)	8 (27%)	19 (44%)	0.201
Abnormal FMD, *n* (%)	5 (17%)	30 (70%)	**0.001**
WBC (10^−3^/*μ*L)	6.20 ± 1.43	7.64 ± 1.73	**0.001**
Hemoglobin (g/dL)	13.46 ± 1.06	12.93 ± 1.78	0.148
Platelet count (10^−3^/*μ*L)	225.50 ± 79.23	250.91 ± 74.67	0.167
MPV (fL)	8.36 ± 0.96	10.47 ± 0.87	**0.001**
Creatinine (mg/dL)	0.85 ± 0.17	0.79 ± 0.21	0.232
AST (IU/L)	21.20 ± 5.14	25.60 ± 9.77	**0.027**
ALT (IU/L)	23.45 ± 7.36	26.12 ± 12.94	0.311
D-dimer (%)	1.25 ± 1.15	2.50 ± 5.049	0.185
Fibrinogen (*μ*g/ml)	258.94 ± 89.74	375.57 ± 160.49	**0.001**
FAR (%)	88.38 ± 66.85	112.54 ± 45.49	0.090
NLR (%)	9.44 ± 11.26	8.07 ± 6.03	0.501
Albumin (g/dL)	3.3500 ± 0.38841	3.3279 ± 0.70561	0.579
DDAR (%)	0.3819 ± 0.37301	0.9210 ± 1.65428	0.877
MCP-1 (pg/ml)	264.4342 ± 71.12647	332.4933 ± 39.45015	**0.001**

FMD: flow-mediated dilatation; WBC: white blood cell; MPV: main platelet volume; AST: aspartate aminotransferase; ALT: alanine aminotransferase; FAR: fibrinogen-to-albumin ratio; NLR: neutrophil-to-lymphocyte ratio; DDAR: D-dimer-to-albumin ratio; MCP-1: monocyte chemoattractant protein-1. ^#^*p* < 0.05 is considered as statistically significant. The bold numbers show the *p* values below 0.05.

**Table 2:** Relation between laboratory parameters and abnormal flow-mediated dilatation (FMD).

	FMD	*p*	
MCP-1	*R*		**0.308**
	*R* ^2^		0.095
	*p*		**0.044**
	*N*		43
WBC	*R*		0.036
	*R* ^2^		0.001
	*p*		0.819
	*N*		43
MPV	*R*		0.171
	*R* ^2^		0.029
	*p*		0.272
	*N*		43
Fibrinogen	*R*		0.101
	*R* ^2^		0.010
	*p*		0.517
	*N*		43

FMD: flow-mediated dilatation; MCP-1: monocyte chemoattractant protein-1; WBC: white blood cell; MPV: mean platelet volume; *p*: correlation is significant at the 0.05 level (2-tailed). The bold numbers show the *p* values below 0.05.

**Table 3:** Multiple binary logistic regression analysis.

	B	SE	Wald	d*f*	Sig.	Exp (B)	95% CI for Exp (B)	
							Lower	Upper
FMD (ref: normal), abnormal	2.202	0.761	8.378	1	0.004	9.039	2.036	40,138
WBC (ref: up to 6.82), 6.82 or higher	1.693	0.730	5.371	1	0.020	5.433	1.298	22.734
MCP (ref: up to 273.30), 273.30 or higher	3.034	0.931	10.611	1	0.001	20.774	3.348	128.896
Constant	−0.066	0.472	0.020	1	0.888	0.936		
(A) Variable (s) entered on step 1: FMD, Kwbc, Kmcp1								
Model summary								
	−2 log likelihood^a^	Cox and Snell *R* square	Nagelkerke's *R* square	Step	Step			
1	51.540	0.477	0.643		1		(a) The cutoff value is 0.500	
Classification table								
Observed						Predicted		
						Groups		Percentage correct
						Control	Raynaud	
Step 1	Groups	Control and Raynaud's				25	5	83.3
						6	37	86.0
Overall percentage								84.9

## Data Availability

The data used to support the findings of this study are included within the article.
